# Supplementary material for: Prioritized polycystic kidney disease drug targets and repurposing candidates from pre-cystic and cystic mouse Pkd2 model gene expression reversion
Source: Mol Med. 2023 May 22;29:67. doi: 10.1186/s10020-023-00664-z (PMC10201779; doi:10.1186/s10020-023-00664-z)
Supplement: Supplementary file 1 — Additional file 1: Summary of Data Sets used in this Study. [file 10020_2023_664_MOESM1_ESM.docx]

**Additional file 1: Summary of Data Sets used in this Study.**

| **Data Origination** | **Kidney Phenotype at Collection** | **Age at Collection (days)** | **Mouse Model** | **Sample Numbers (KO/WT)** |
| --- | --- | --- | --- | --- |
| GSE149739 [(Zhang et al., 2021)](https://paperpile.com/c/aDmF9M/sKTsP) | Pre-cystic, mild tubule dilation but no overt cyst formation | 70 | *Pkd2^fl^*^/fl^;*Pax8*rtTA;TetO-Cre | 3/3 |
| GSE134719 [(Lee et al., 2019)](https://paperpile.com/c/aDmF9M/3v6Uy) | Cystic | 28 | *Pkhd1*-Cre; *Pkd2^fl^*^/fl^ | 11/12 |
| GSE69556 [(Lakhia et al., 2016)](https://paperpile.com/c/aDmF9M/x93vS) | Cystic | 21 | *Pkhd1*-Cre; *Pkd2^fl^*^/fl^ | 3/4 |

GEO accession numbers for publicly available data used in this study along with kidney phenotype and age at collection, mouse model genotype, and number of samples used from each data set.
