# Supplementary material for: Prioritized polycystic kidney disease drug targets and repurposing candidates from pre-cystic and cystic mouse Pkd2 model gene expression reversion
Source: Mol Med. 2023 May 22;29:67. doi: 10.1186/s10020-023-00664-z (PMC10201779; doi:10.1186/s10020-023-00664-z)
Supplement: Supplementary file 2 — Additional file 2: Pathway Enrichment of Pre-cysticvs CysticKidney Differential Gene Expression. Bubbleplots showing enrichment for genes uniquely differentially expressed in the A) pre-cystic data set and B) both cystic data sets, where point size is the ratio of query genes to pathway gene set size. [file 10020_2023_664_MOESM2_ESM.docx]

**Additional file 2: Enrichment of Unique Pre-Cystic DEGs and Overlapping Cystic Differentially Expressed Genes**

**
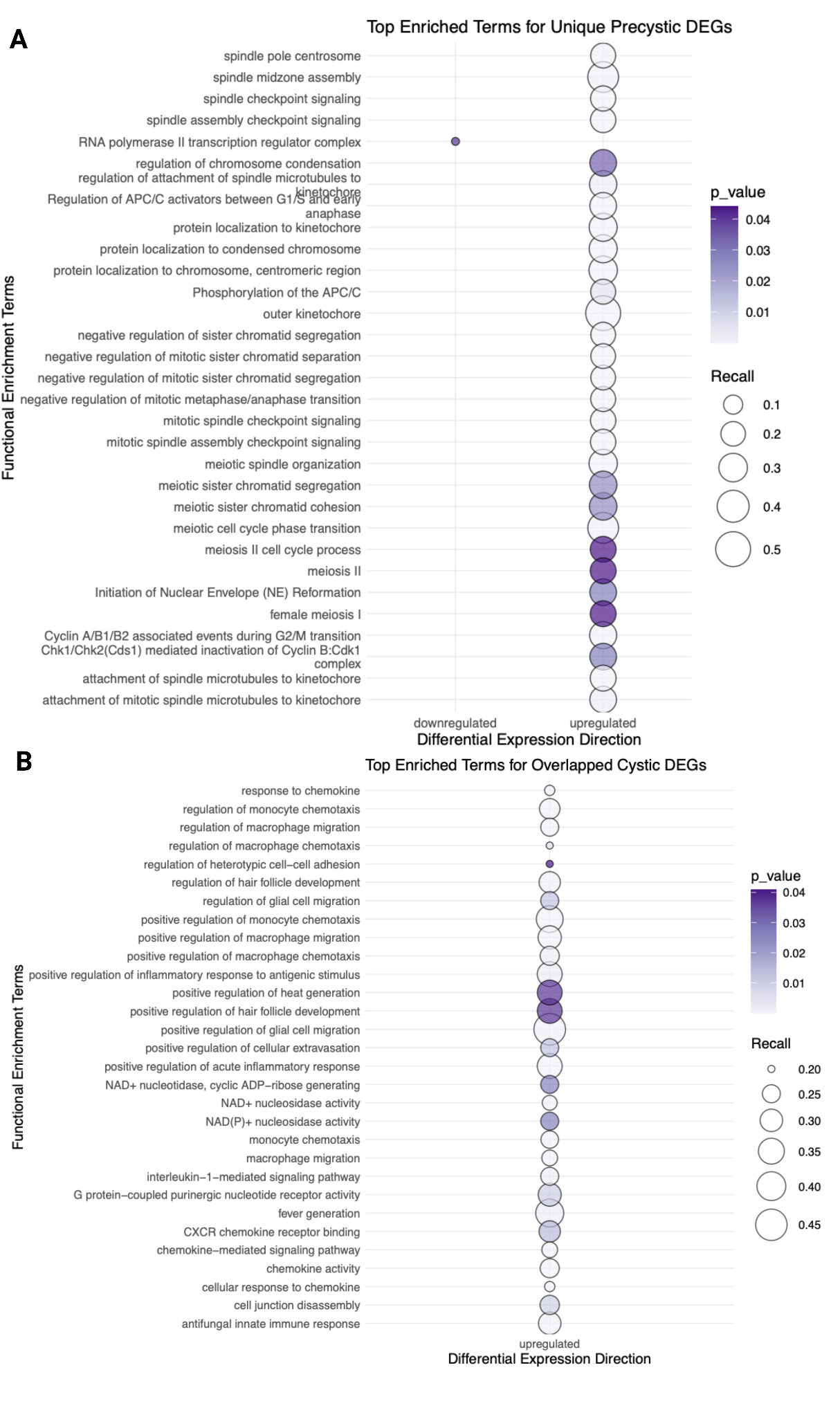
**

**Additional file 2: Pathway Enrichment of Pre-cystic (P70) vs Cystic (P21 and P28) Kidney Differential Gene Expression** Bubbleplots showing enrichment for genes uniquely differentially expressed in the A) pre-cystic data set and B) both cystic data sets, where point size is the ratio of query genes to pathway gene set size.
