# Supplementary material for: Prioritized polycystic kidney disease drug targets and repurposing candidates from pre-cystic and cystic mouse Pkd2 model gene expression reversion
Source: Mol Med. 2023 May 22;29:67. doi: 10.1186/s10020-023-00664-z (PMC10201779; doi:10.1186/s10020-023-00664-z)
Supplement: Supplementary file 3 — Additional file 3: Differential Expression Analysis. Treemaps of A) pre-cystic P70, B) cystic P21, and C) cystic P28 differentially expressed genes display enrichment nesting, with GO:BP parent terms consisting of largest boxes and enriched child terms reflecting enrichment term size nested within, for each signature. [file 10020_2023_664_MOESM3_ESM.docx]

**Additional file 3: Pathway Enrichment for Differentially Expressed Genes**

**
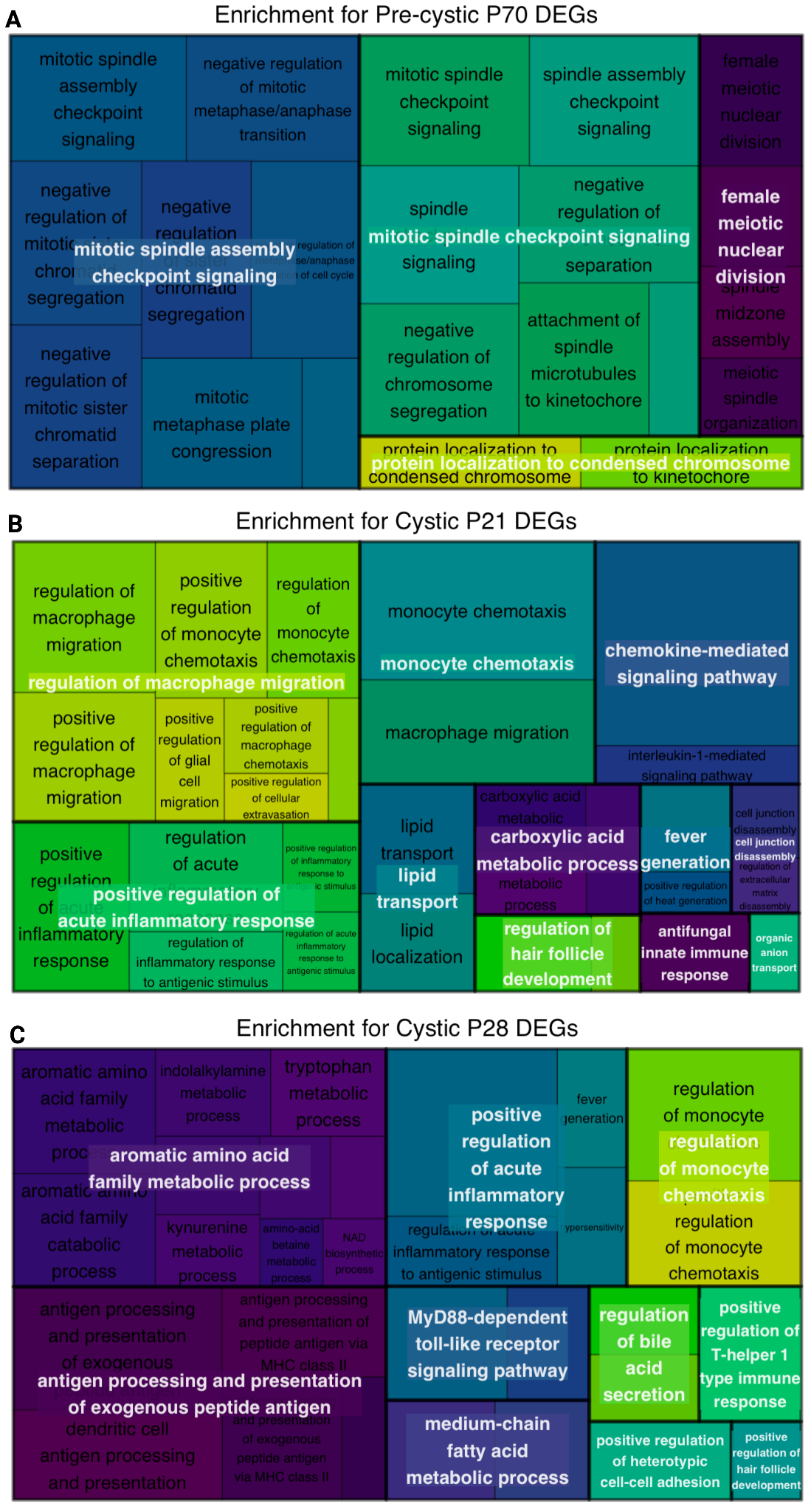
**

**Additional file 3: Differential Expression Analysis** Treemaps of A) pre-cystic P70, B) cystic P21, and C) cystic P28 differentially expressed genes display enrichment nesting, with GO:BP parent terms consisting of largest boxes and enriched child terms reflecting enrichment term size nested within, for each signature.
