# Supplementary material for: Prioritized polycystic kidney disease drug targets and repurposing candidates from pre-cystic and cystic mouse Pkd2 model gene expression reversion
Source: Mol Med. 2023 May 22;29:67. doi: 10.1186/s10020-023-00664-z (PMC10201779; doi:10.1186/s10020-023-00664-z)
Supplement: Supplementary file 5 — Additional file 5: Transcriptomic Signatures for Pre-cystic and Cystic Disease Signature Reversion. Heatmaps of rlog gene expression counts for genes selected for transcriptomic reversion signaturesshowing complete linkage hierarchical clustering of gene expression and samples for A) pre-cystic P70, B) cystic P21, and C) cystic P28. D) Venn diagram of transcriptomic reversion signatures from the 3 data sets showing overlap and unique genes for each signature. [file 10020_2023_664_MOESM5_ESM.docx]

**Additional file 5: Transcriptomic Signatures for Pre-cystic and Cystic Disease Signature Reversion.**


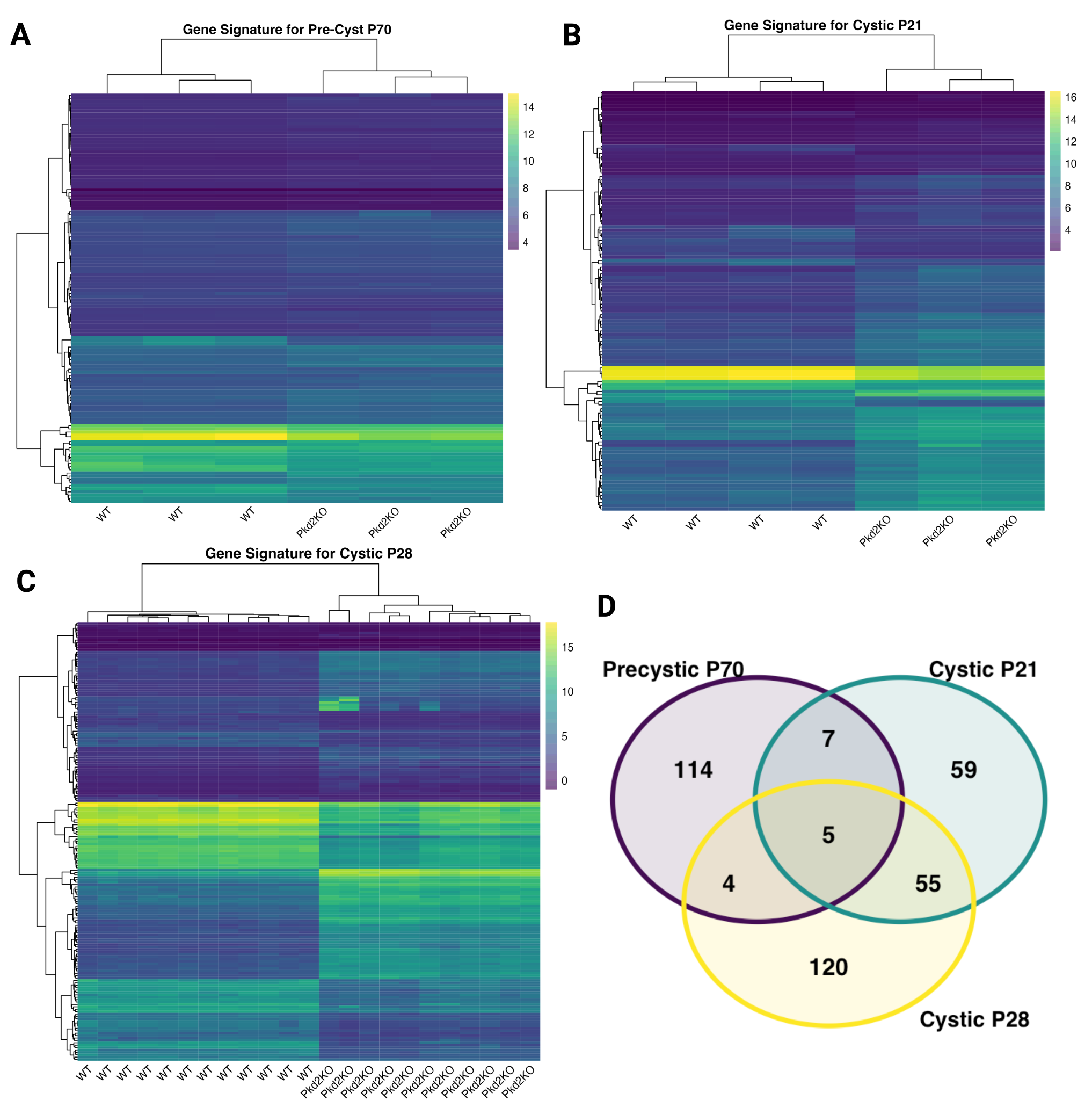


**Additional file 5: Transcriptomic Signatures for Pre-cystic and Cystic Disease Signature Reversion.** *Heatmaps of rlog gene expression counts for genes selected for transcriptomic reversion signatures (filtered by genes available in LINCS perturbation data and then top 100-200 absolute LFC genes) showing complete linkage hierarchical clustering of gene expression and samples for A) pre-cystic P70, B) cystic P21, and C) cystic P28. D) Venn diagram of transcriptomic reversion signatures from the 3 data sets showing overlap and unique genes for each signature.*
